# Comparative outcomes of on-label and off-label transcatheter aortic valve replacement for aortic regurgitation: a systematic review and meta-analysis

**DOI:** 10.1136/openhrt-2025-003482

**Published:** 2025-10-17

**Authors:** Yanren Peng, Yongqing Lin, Zizhuo Su, Shen Nie, Ruqiong Nie, Yangxin Chen

**Affiliations:** 1 Department of Cardiovascular Medicine, Sun Yat-Sen Memorial Hospital, Guangzhou, Guangdong, China

**Keywords:** Meta-Analysis, Aortic Valve Insufficiency, Outcome Assessment, Health Care

## Abstract

**Background:**

Transcatheter aortic valve replacement (TAVR) has emerged as an alternative treatment for aortic regurgitation (AR) in patients at high surgical risk. However, evidence comparing outcomes of different new-generation devices remains limited.

**Objectives:**

To compare clinical outcomes of on-label, off-label self-expanding (SE) and off-label balloon-expandable (BE) TAVR devices in AR patients.

**Methods:**

A systematic review and meta-analysis were conducted, including studies reporting clinical outcomes of new-generation TAVR devices in AR. The primary outcome was 1-year all-cause mortality. Secondary outcomes included procedural success, moderate or severe AR and perioperative complications. Subgroup and meta-regression analyses assessed the impact of valve type and clinical variables.

**Results:**

32 studies involving 2682 patients were included. 1-year mortality was 10.4% (95% CI: 7.2% to 14.7%) with no significant difference among valve types. Technical success was highest with on-label devices (97%), followed by off-label:BE (92%) and off-label:SE (85%) (p<0.001). Valve migration occurred in 2% of on-label, 7% of off-label:BE and 10% of off-label:SE cases (p=0.004). Moderate or severe AR was observed in 2% of on-label, 4% of off-label:BE and 8% of off-label:SE recipients (p<0.001). Meta-regression identified coronary heart disease as an independent predictor of 1 year mortality (p=0.026), while other factors showed no significant association.

**Conclusions:**

On-label devices were associated with improved procedural outcomes, including lower rates of valve migration and residual AR, although 1-year mortality did not differ significantly between device groups. Further prospective studies with longer follow-up are needed to assess valve durability and long-term clinical outcomes.

**PROSPERO registration number:**

CRD 42024611296.

**WHAT IS ALREADY KNOWN ON THIS TOPIC**

- Transcatheter aortic valve replacement (TAVR) is an alternative treatment for aortic regurgitation (AR) but faces challenges due to anatomical factors, such as the lack of calcification, complicating valve anchoring and procedural success.

**WHAT THIS STUDY ADDS**

- This study demonstrates superior procedural outcomes for on-label TAVR devices compared with off-label devices, particularly with significantly lower rates of valve migration and residual AR. Among off-label devices, balloon-expandable valves required significantly fewer second valve implantations compared with self-expanding valves, although other complication rates were similar.

**HOW THIS STUDY MIGHT AFFECT RESEARCH, PRACTICE OR POLICY**

- These findings support preferential selection of on-label devices in AR patients and inform clinicians about differences in off-label device performance, highlighting the need for prospective studies evaluating long-term outcomes and valve durability.

## Introduction

 Aortic regurgitation (AR) is the third most common valvular heart disease worldwide, affecting approximately 4.5% of individuals aged 65 years and older.^1 2^ Moderate to severe AR is associated with significantly reduced survival compared with age-matched and sex-matched controls, with prognosis worsening alongside New York Heart Association (NYHA) classification. In patients with NYHA III-IV symptoms, the 4-year survival rate is as low as 28%.^3 4^ However, despite its adverse prognosis, the intervention rate for severe AR remains low, with fewer than one-third of eligible patients undergoing treatment. In those with left ventricular ejection fraction (LVEF) <30%, the intervention rate drops to below 5%.^5 6^

TAVR has emerged as an alternative for AR patients deemed high-risk or ineligible for surgical aortic valve replacement (SAVR). However, unlike aortic stenosis, where leaflet calcification facilitates valve anchoring, AR presents unique procedural challenges, including larger annular dimensions, strong regurgitant jets and the absence of calcified landing zones. These factors contribute to higher rates of valve migration and paravalvular leak (PVL), impacting procedural success.^7^ Early-generation TAVR devices, such as Sapien XT and CoreValve, exhibited limited efficacy in AR patients, but newer-generation valves have shown improved technical success and reduced 1-year cardiovascular mortality.^8 9^

New-generation TAVR devices are categorised as on-label or off-label, depending on regulatory approval for AR (online supplemental table 1). Off-label devices are further classified into self-expanding (SE) and balloon-expandable (BE) valves. Off-label:BE devices (eg, Sapien 3, Myval) are deployed via balloon inflation, achieving anchoring primarily at the annulus.^10 11^ In contrast, off-label:SE devices (eg, Evolut PRO, Acurate neo) gradually expand and typically feature a flared structure, allowing anchoring at both the annulus and the sinotubular junction or ascending aorta.^12 13^ On-label devices, such as JenaValve and J-Valve, incorporate U-shaped anchoring claspers, enabling dual fixation at both the leaflet and annulus, thereby improving fixation and reducing PVL.^14 15^

Despite advancements in valve technology, evidence on TAVR for AR remains limited, with existing studies constrained by small sample sizes and heterogeneous patient populations. To date, no study has directly compared 1-year mortality across different new-generation TAVR devices, nor has any analysis comprehensively assessed the clinical outcomes of on-label, off-label SE and off-label BE valves in AR patients. Therefore, we performed a systematic review and meta-analysis to evaluate the comparative effectiveness and clinical outcomes of new-generation TAVR devices in AR, with a specific focus on 1-year mortality.

## Methods

### Literature search

We conducted a systematic literature search in MEDLINE, Embase, the Cochrane Central Register of Controlled Trials (CENTRAL) and Web of Science through 8 October 2024, using predefined search terms (online supplemental table 2). The study protocol was registered in PROSPERO (CRD 42024611296). Two independent reviewers (YP, YL) screened titles and abstracts for relevance. Full-text screening was performed for eligible studies, and reference lists were manually searched to identify additional reports.

### Study selection

Studies were eligible if they met the following criteria: (1) included patients with pure native aortic valve regurgitation who underwent TAVR; (2) had a minimum sample size of 20 patients; (3) reported baseline patient characteristics, primary and secondary outcome data; (4) were published in English and (5) excluded abstracts, conference presentations, case reports, editorials, letters, reviews and meta-analyses.

To ensure the inclusion of only new-generation TAVR devices, studies evaluating first-generation devices (eg, CoreValve (Medtronic, Minneapolis, Minnesota, USA) and SAPIEN XT (Edwards Lifesciences, Irvine, California, USA)) were excluded. The analysis focused on second-generation TAVR devices, collectively referred to as new-generation devices (NGDs), including JenaValve, J-Valve, SAPIEN 3, Evolut R, Direct Flow, ACURATE and Lotus. On-label devices comprised J-Valve and JenaValve, which were specifically approved for AR, while all other NGDs were categorised as off-label. If multiple reports described overlapping patient populations, the most recent and comprehensive study was retained.

### Data extraction

Data extraction was performed using a predefined collection form, including the first author, year of publication, number of patients and device type. Collected variables included patient demographics (age, sex, comorbidities), clinical and procedural characteristics (vascular access, procedural success, valve-in-valve, permanent pacemaker implantation (PPI) and mortality) and echocardiographic and structural data (LVEF, left ventricular end-diastolic diameter and annulus perimeter). Additional extracted details encompassed study design, geographic region, study period and Valve Academic Research Consortium definitions. Two independent reviewers (YP, YL) conducted data extraction to ensure accuracy.

### Outcomes of interest

The primary outcome was 1-year all-cause mortality. Secondary outcomes included in-hospital and 30-day mortality, procedural success (technical and device success), moderate or severe AR, valve embolisation or migration, NYHA functional class III/IV, acute kidney injury (AKI), PPI and stroke. Procedural complications evaluated included major bleeding, major vascular complications and blood transfusion requirements. Additional endpoints such as myocardial infarction, conversion to surgery, coronary obstruction and annular rupture were also analysed.

### Risk of bias assessment

The risk of bias in randomised controlled trials (RCTs) was assessed using the Cochrane Risk of Bias Tool, while non-randomised studies were evaluated using the Methodological Index for Non-Randomized Studies criteria (online supplemental table 8). Publication bias was assessed using funnel plots and Egger’s regression analysis to detect small-study effects.

### Data synthesis and analysis

Categorical variables were expressed as proportions with 95% CIs and continuous variables as means±SDs or medians (IQRs). Heterogeneity was quantified using the I² statistic, with *I*² ≥ 50% indicating substantial heterogeneity. Statistical significance was set at p value<0.05.

Meta-analyses were conducted using the meta and metafor packages in R V.4.4.2 (R Foundation for Statistical Computing). Event rates were summarised using the metaprop function with logit transformation (PLOGIT) to stabilise variance, and zero-event studies were adjusted by adding 0.5 events and 1 to the total sample size.

Subgroup analyses were performed based on valve type (on-label, off-label:SE, off-label:BE). Between-group differences were assessed using Cochran’s Q-test, and pairwise comparisons were adjusted for multiple testing using the false discovery rate (FDR) method. In addition, four predefined sensitivity analyses (SA) were conducted to assess the robustness of findings: SA-1, excluding studies with mean Society of Thoracic Surgeons (STS) score >8%; SA-2, studies in which ≥90% of patients underwent transfemoral access; SA-3, leave-region-out analyses excluding Asian, European or North American cohorts in turn and SA-4, excluding small studies (<30 patients) reporting a single valve platform. To explore potential effect modifiers, meta-regression analyses were conducted using the rma function, evaluating predictors of 1-year mortality, with p values adjusted for FDR.

## Results

### Study selection and study characteristics

**Figure 1:**
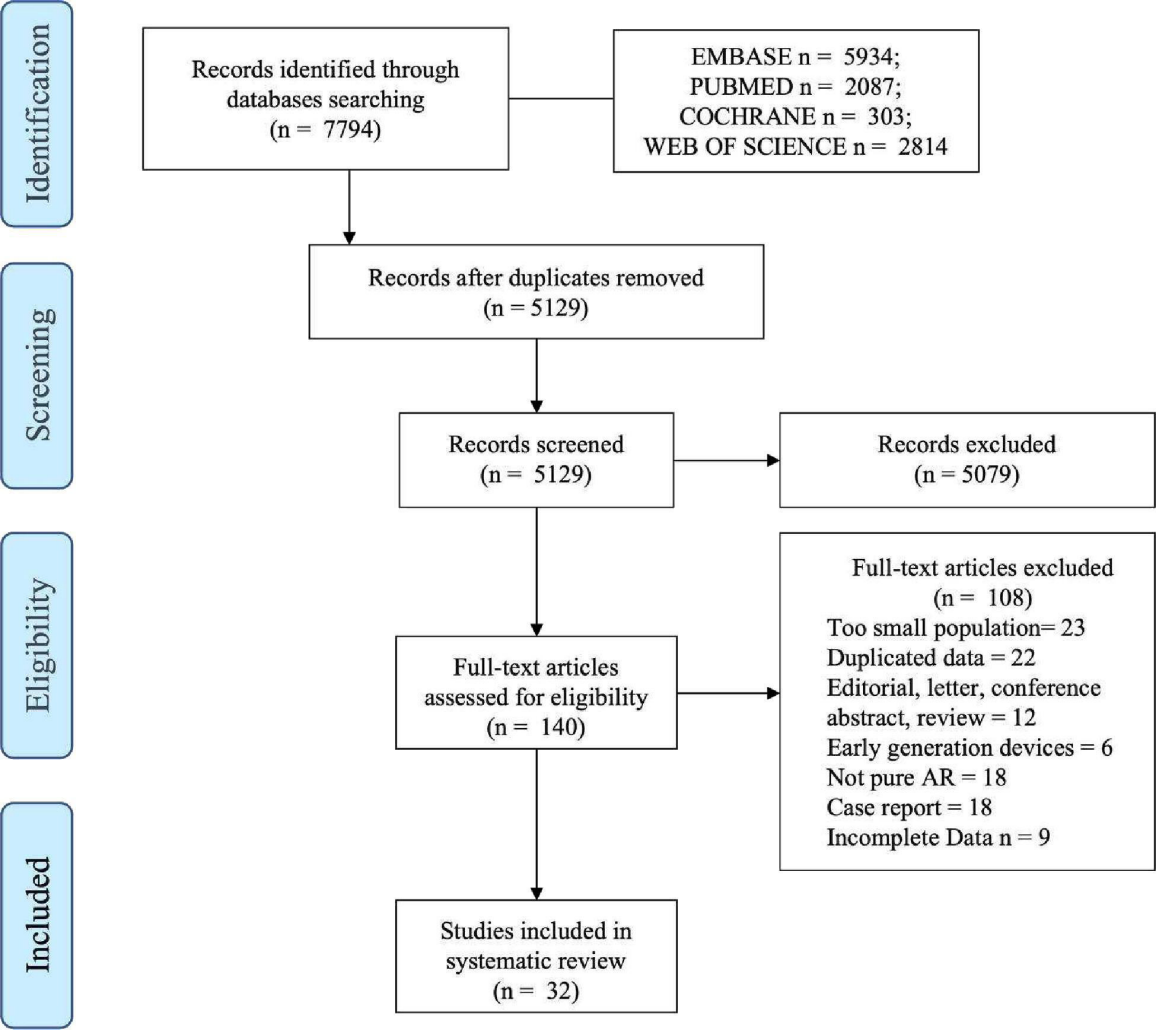
Flow diagram of manuscript selection according to Preferred Reporting Items for Systematic Reviews and Meta-Analyses statement. AR, aortic regurgitation.

A total of 7794 articles were initially identified through comprehensive database searches. After removing duplicates, 5129 articles underwent preliminary evaluation based on their titles and abstracts. Subsequently, 140 articles were selected for full-text review. Following rigorous assessment, 32 studies met the predefined inclusion criteria and were incorporated into the final meta-analysis. The selection process is illustrated in the Preferred Reporting Items for Systematic Reviews and Meta-Analyses flow diagram (figure 1).

A total of 2682 patients were analysed, with a mean age of 73.1±9.1 years and 39.1% were female. The mean STS score was 6.1±4.8, and the average LVEF was 49.1±12.9%. The study population included 963 on-label cases, 294 off-label:BE cases, 773 off-label:SE and 652 ungrouped pure AR cases. Study characteristics are summarised in (table 1, online supplemental table 3–5.)

**Table 1:** Main clinical and procedural characteristics of patients in included studies

	N	Age (years)	Female (%)	STS score (%)	Access	Device	Device success (%)	ViV (%)	PPI (%)	30-day mortality (%)	1-year mortality (%)
Kong *et al*^23^	69	71.5±7.9	24.6	3.8±3.9	Apical	J-Valve	–	–	7.2	1.5	–
Chen *et al*^24^	77	78.1±12.5	52	7.7±5.9	Femoral	VenusA-Valve, VitaFlow	80.5	19.5	11.7	–	–
Tung et al^25^	43	73.8±5.7	30.2	–	Apical	J-Valve	95.3	0	5	4.7	5
Wang *et al*^26^	61	72.8±6.7	37.7	5.8±1.8	Femoral 98.4% Others 1.6%	Venus-A Venus-A Plus	73.8	21.3	19.7	3.3	–
Chen *et al*^27^	37	73.1±8.7	37.8	8.6±2.1	Femoral	–	67.6	21.6	24.3	0	5.4
Sanchez-Luna *et al*^11^	113	78.4±7.46	35.4	2.7±1.7	Femoral	–	94.7	3.5	13.4	5.3	9.7
Yang *et al*^28^	55	71.2±8.7	61.8	3.4±1.9	Femoral	–	80	20	27.3	3.6	–
Yu *et al*^29^	30	70.4±8.4	26.7	–	Femoral	Venus-A valve	66.7	–	–	–	–
Liu *et al*^30^	161	72.5±6.2	26.1	9.9±5.7	Apical	J-Valve	95	0.6	8.3	1.9	–
Seiffert *et al*^31^	31	73.8±9.1	35.5	5.4±3.6	Apical	JenaValve	96.8	3.2	6.4	12.9	–
Le Ruz *et al*^32^	227	81 (73.5–85.0)	35.7	–	Femoral 91.6% Others 8.4%	Evolut PRO Evolut R, SAPIEN 3	–	8.8	–	8.4	24.2
Poletti *et al*,^33^ BE-PANTHEON	144	–	33.3	2.7 (1.9–4.6)	Femoral 99.3% Others 0.7%	MyVal, Sapien	81	6.9	13.1	4.2	7.3
Poletti *et al*,^34^ PURPOSE	88	80.0 (72.0–84.0)	40.9	2.6 (1.8–4.4)	Femoral	JenaValve Trilogy	95	1.1	24	1.1	–
Tsai *et al*^14^	68	80.0 (71.0–84.0)	38.2	2.5 (1.8–4.3)	Femoral	JenaValve Trilogy	–	–	16.4	–	–
*Sawaya et al* ^35^	41	72±10	42	5.6±3.8	Femoral 44% Apical 56%	Evolut R, JenaValve, Direct Flow, Lotus System, Sapien 3	85	10	–	8	–
Anwaruddin *et al*^36^	230	68.9±15.1	46	8.6±9.9	–	Evolut R	86.9	–	20	10	–
Kong *et al*^37^	62	71.6±7.3	41.9	5.4±3.2	Femoral	VitaFlow	79	14.5	29	–	6.5
Silaschi *et al*^38^	30	74.4±9.3	60	4.9±3.5	Apical	JenaValve	88.9	0	3.8	10	20
Liu *et al*^39^	47	73.7±7.9	27.7	–	Apical	J-Valve	–	2.1	0	2.1	2.1
Mao *et al*^40^	125	72.5±5.6	29.6	8.3±3.5	Apical	J-Valve	–	80	3.2	–	–
Jin *et al*^41^	36	74 (68.5–79.0)	36.1	3.9 (2.3–5.7)	Apical	J-Valve	–	–	–	2.8	2.9
Delhomme *et al*^10^	37	81 (69–85)	27	–	Femoral 94.6% Apical 2.7% Others 2.7%	SAPIEN 3	–	2.7	35.1	8.1	16.2
Adam *et al*^42^	58	76.5±9.0	36.2	4.2±4.3	Femoral	JenaValve Trilogy	98	0	19.6	1.7	–
Poletti et al,^43^ PANTHEON	132	79 (72–83)	52.3	5.2 (4.3–6.2)	Femoral 93.4% Others 6.6%	–	75.8	11.4	22.6	–	–
Yoon *et al*^8^	212	74.5±11.6	50.9	6.2±6.7	Femoral 60.8% Apical 35.8% Others 3.4%	Evolut R, Sapien 3, JenaValve, Lotus, Direct Flow, Acurate, Portico, J-Valve,	81.1	12.7	18.6	9.4	20.6
Hinkov *et al*^44^	27	65.3 (36.8–77.6)	33.3	–	Femoral 92.6% Others 7.4%	JenaValve Trilogy Sapien 3	–	22.2	22.2	–	–
Garcia *et al*^45^	27	81 (72–85)	41	4.3 (2.6–5.3)	Femoral 78%, Others 22%	J-Valve System	–	11.1	12.5	4.2	11.8
Purita *et al*^46^	24	79.4 (^51–88^)	58.4	3.9±2.4	Femoral	Acurate neo	87.5	12.5	21.1	4.2	11.8
Toggweiler *et al*^47^	20	79±8	75	8.3±9.3	Femoral	Acurate neo	90	10	15	0	–
Zheng *et al*^48^	45	73.5±5.5	26.7	–	Femoral	Venus A-Valve	95.6	0	0	–	4.7
De Backer *et al*^9^	145	75±10	51	6.2±4.9	Femoral 76% Apical 18% Others 6%	EvolutR, Sapien 3, Engager, JenaValve, Portico, Acurate, Lotus, Direct Flow	82	–	–	8	–
Vahl *et al*^49^	180	75.5±10.8	47	4.1±3.4	Femoral	JenaValve Trilogy	–	1.7	24	2.2	8

PPI, permanent pacemaker implantation; STS, Society of Thoracic Surgeons score; ViV, valve in valve.

### Meta-Analysis of clinical outcomes

**Figure 2:**
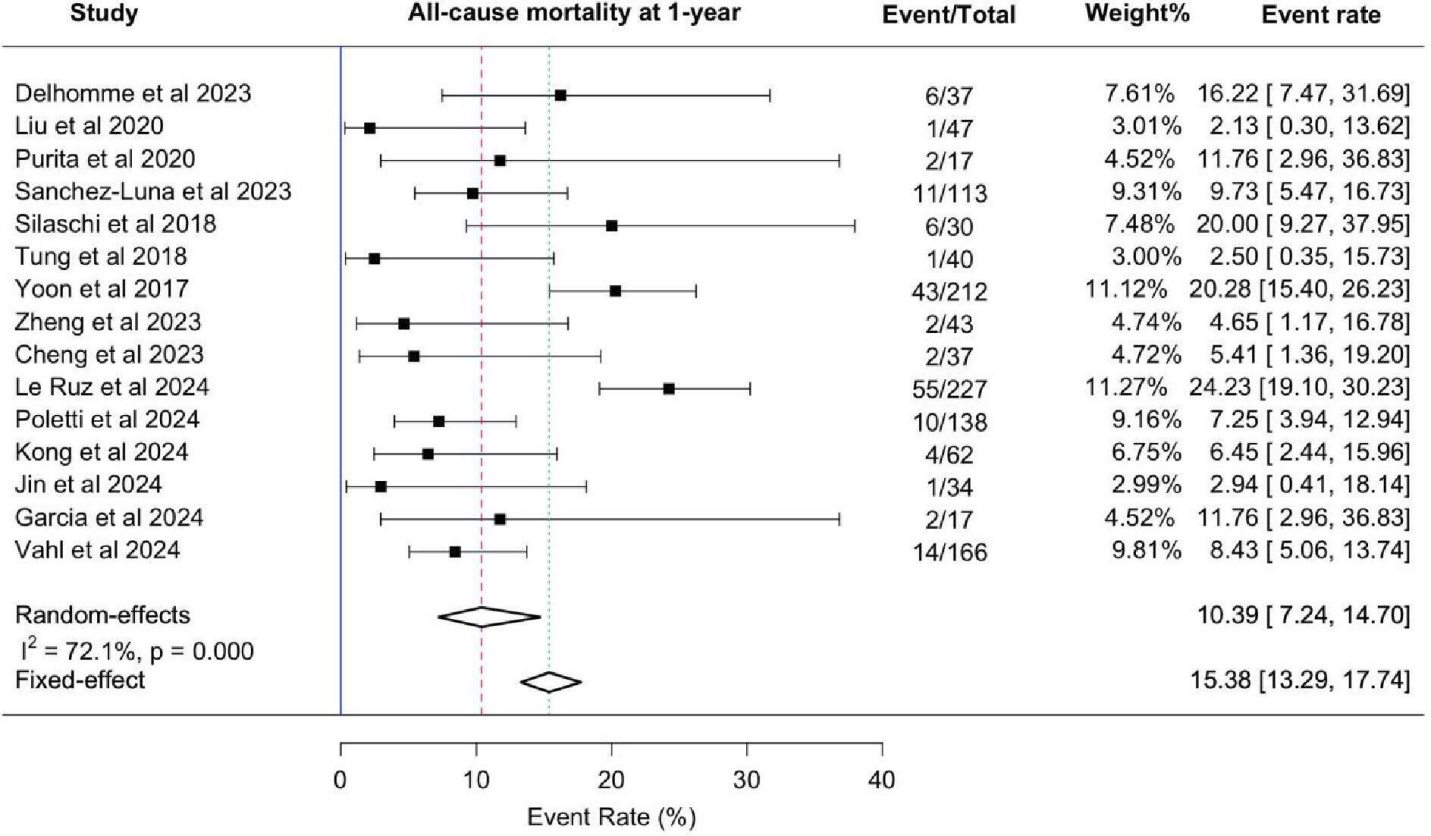
Forest Plot Showing the Individual and Pooled Event Rates for the Primary Endpoint, t plot showing the individual and pooled event rates for the primary endpoint, 11-year Mortality A mortality after TAVR. A total of 15 studies encompassing 1220 patients were included in this analysis. Event rates were calculated using the DerSimonian-Laird random-effects model with logit transformation to stabilise variance. Each square represents the event rate for an individual study, with the size proportional to its weight in the meta-analysis; horizontal lines indicate 95% CIs. The diamond represents the pooled event rate. Analyses included all eligible new-generation TAVR devices, regardless of label status. TAVR, transcatheter aortic valve replacement.

**Figure 3:**
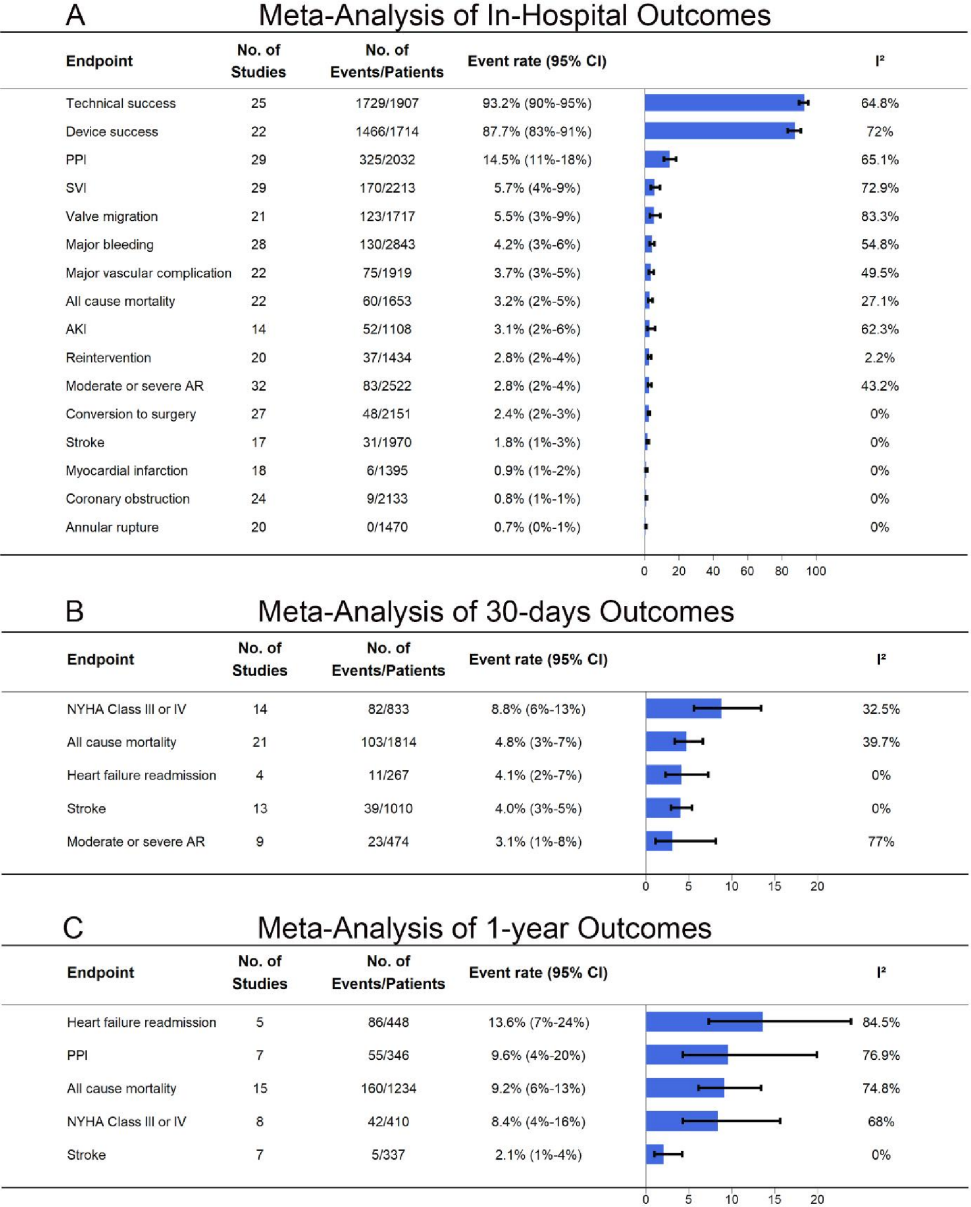
Meta-Analysis of Clinical Outcomes. AR, aortic regurgitation; AKI, acute kidney injury; NYHA, New York Heart Association; PPI, permanent pacemaker implantation; SVI, second valve implantation.

At the 1-year follow-up, the all-cause mortality rate was 10.4% (95% CI: 7.2% to 14.7%) (figure 2). Heart failure rehospitalisation occurred in 13.6% (95% CI: 7.0% to 24.0%), indicating that some patients required continued medical management. PPI remained necessary in 9.6% (95% CI: 4.0% to 20.0%), while NYHA Class III–IV symptoms persisted in 8.4% (95% CI: 4.0% to 16.0%), suggesting symptomatic improvement in most patients. The stroke rate at 1 year was 2.1% (95% CI: 1.0% to 4.0%) (figure 3C).

At 30 days, the mortality rate was 4.8% (95% CI: 3% to 7%), while heart failure rehospitalisation occurred in 4.1% (95% CI: 2% to 7%) (figure 3B). Stroke was reported in 4.0% (95% CI: 3% to 5%), with minimal heterogeneity. Moderate or severe AR persisted in 3.1% (95% CI: 1% to 8%), while 8.8% (95% CI: 6% to 13%) of patients remained in NYHA Class III–IV (figure 3B).

During hospitalisation, technical success was achieved in 93.2% (95% CI: 90% to 95%) and moderate-to-severe AR was reported in 2.8% (95% CI: 2% to 4%) (figure 3A). PPI was required in 14.5% (95% CI: 11% to 18%), while valve migration occurred in 5.5% (95% CI: 3% to 9%). Major vascular complications were observed in 3.7% (95% CI: 2% to 5%) and major bleeding in 4.2% (95% CI: 3% to 6%). In-hospital mortality was 3.2% (95% CI: 2% to 5%), while conversion to surgery was required in 2.4% (95% CI: 2% to 3%)(figure 3A).

### Subgroup analysis: on-label versus off-label SE and BE devices

The results of the subgroup analysis are summarised in table 2. A total of 21 studies (1467 patients) reported technical success. The highest success rate was observed in on-label devices (97%), followed by off-label: BE (92%) and off-label: SE (85%) (p<0.001). Device success, assessed in 19 studies (1316 patients), showed a similar trend (95% vs 89% vs 83%, p<0.001). However, pairwise comparisons did not reveal significant differences after multiple testing corrections.

**Table 2:** Subgroup analysis of clinical outcomes in on-label devices, off-label SE and off-label BE: a meta-analysis

	On-label devices			Off-label SE				Off-label BE			
Outcomes	Event rate (95% CI)	*I*^2^ (%)	P value*	Event rate (95% CI)	*I*^2^ (%)	P value*	Event rate (95% CI)	*I*^2^ (%)	P value*	*χ^2^*	P value†
In-hospital Outcomes											
All cause mortality	0.02 (0.01 to 0.03)	4.7	0.398	0.04 (0.02 to 0.08)	11	0.346	0.04 (0.02 to 0.07)	0	0.797	5.52	0.063
Technical success	0.97 (0.94 to 0.98)	11.8	0.329	0.85 (0.78 to 0.90)	26.7	0.234	0.92 (0.88 to 0.95)	21.4	0.280	17.46	0.000
Device success	0.95 (0.92 to 0.97)	0	0.598	0.83 (0.76 to 0.87)	66.6	0.001	0.89 (0.76 to 0.96)	88.9	0.003	19.75	0.000
PPI	0.10 (0.06 to 0.15)	74.3	0.000	0.19 (0.14 to 0.24)	34.3	0.133	0.18 (0.10 to 0.29)	80.7	0.006	6.18	0.046
Moderate or severe AR	0.02 (0.01 to 0.03)	0	0.745	0.04 (0.02 to 0.07)	31.6	0.156	0.08 (0.06 to 0.12)	1.1	0.364	27.05	0.000‡§
SVI	0.02 (0.01 to 0.03)	18.4	0.269	0.15 (0.11 to 0.21)	46.2	0.053	0.05 (0.03 to 0.08)	0	0.382	50.92	0.000‡§¶
Valve migration	0.02 (0.01 to 0.04)	10.8	0.346	0.10 (0.05 to 0.22)	84.7	0.000	0.07 (0.04 to 0.11)	42.1	0.178	10.89	0.004‡§
Major bleeding	0.04 (0.02 to 0.06)	0.2	0.439	0.05 (0.03 to 0.09)	74.2	0.000	0.03 (0.01 to 0.08)	76.9	0.005	0.86	0.651
AKI **2 or 3**	0.02 (0.01 to 0.04)	0	0.647	0.04 (0.01 to 0.17)	50.1	0.138	0.03 (0.01 to 0.18)	81.9	0.019	57.9	0.006
30-days Outcomes											
All cause mortality	0.03 (0.02 to 0.05)	41.7	0.080	0.06 (0.03 to 0.11)	20.3	0.285	0.06 (0.03 to 0.11)	0	0.537	4.14	0.162
Stroke	0.02 (0.01 to 0.05)	0	0.938	0.05 (0.03 to 0.07)	0	0.938	–	–	–	2.07	0.151
Moderate or severe AR	0.01 (0.00 to 0.03)	0	0.870	0.09 (0.03 to 0.23)	75.1	0.007	–	–	–	8.02	0.005
1-year Outcomes											
All cause mortality	0.07 (0.03 to 0.13)	48.6	0.083	0.09 (0.03 to 0.11)	0	0.787	0.09 (0.07 to 0.13)	25.5	0.261	1.67	0.434
Heart failure readmission	–	–	–	0.08 (0.03 to 0.16)	0	0.470	0.11 (0.06 to 0.22)	72.8	0.055	0.61	0.436
NYHA Class III or IV	0.16 (0.02 to 0.63)	29.9	0.211	0.10 (0.02 to 0.43)	84	0.012	–	–	–	0.15	0.926
PPI	0.06 (0.03 to 0.12)	0	0.977	0.11 (0.01 to 0.54)	88	0.004	–	–	–	0.087	0.341

* test for heterogeneity;

† test for subgroup differences;

‡ On-label devices versus off-label SE;

§ on-label devices versus off-label BE;

¶ off-label SE versus off-label BE;

AKI, acute kidney injury; AR, aortic regurgitation; BE, balloon-expandable; NYHA, New York Heart Association; PPI, permanent pacemaker implantation; SE, self-expanding; SVI, second valve implantation.

For moderate or severe AR during hospitalisation, 27 studies (1908 patients) were analysed. The incidence was significantly lower in on-label devices (2%) compared with off-label SE (8%, adjusted p=0.006) and off-label BE (4%, adjusted p=0.012). No significant difference was found between off-label SE and BE devices (adjusted p=0.232).

Valve migration, analysed in 20 studies (1572 patients), was more frequent in off-label:SE (10%) compared with off-label:BE (7%) and on-label devices (2%) (p=0.004). Pairwise comparisons showed that valve migration was significantly higher in off-label SE compared with on-label devices (adjusted p=0.020) but not between off-label SE and BE (adjusted p=0.510). Similarly, off-label BE had a significantly higher valve migration rate than on-label devices (adjusted p=0.020).

Second valve implantation (SVI) was assessed in 24 studies (1695 patients). SVI was most frequent in off-label:SE devices (15% (95% CI: 11% to 21%)), significantly higher than in on-label devices (2% (95% CI: 1% to 3%), adjusted p<0.001) and off-label BE devices (5% (95% CI: 3% to 8%), adjusted p=0.013). Off-label BE devices also had a significantly higher SVI rate than on-label devices (adjusted p=0.042).

At 30 days, data from eight studies (440 patients) showed that moderate or severe AR was significantly more frequent in off-label:SE devices (9% (95% CI: 3% to 23%)) compared with on-label devices (1% (95% CI: 0% to 3%), p=0.005). At 1 year, analysis of eight studies (437 patients) revealed that NYHA Class III–IV symptoms were observed in 22% (95% CI: 2% to 79%) of on-label devices, significantly higher than in off-label:SE devices (10% (95% CI: 2% to 43%), p=0.012).

### Comparison of on-label vs off-label devices for mortality outcomes

**Figure 4:**
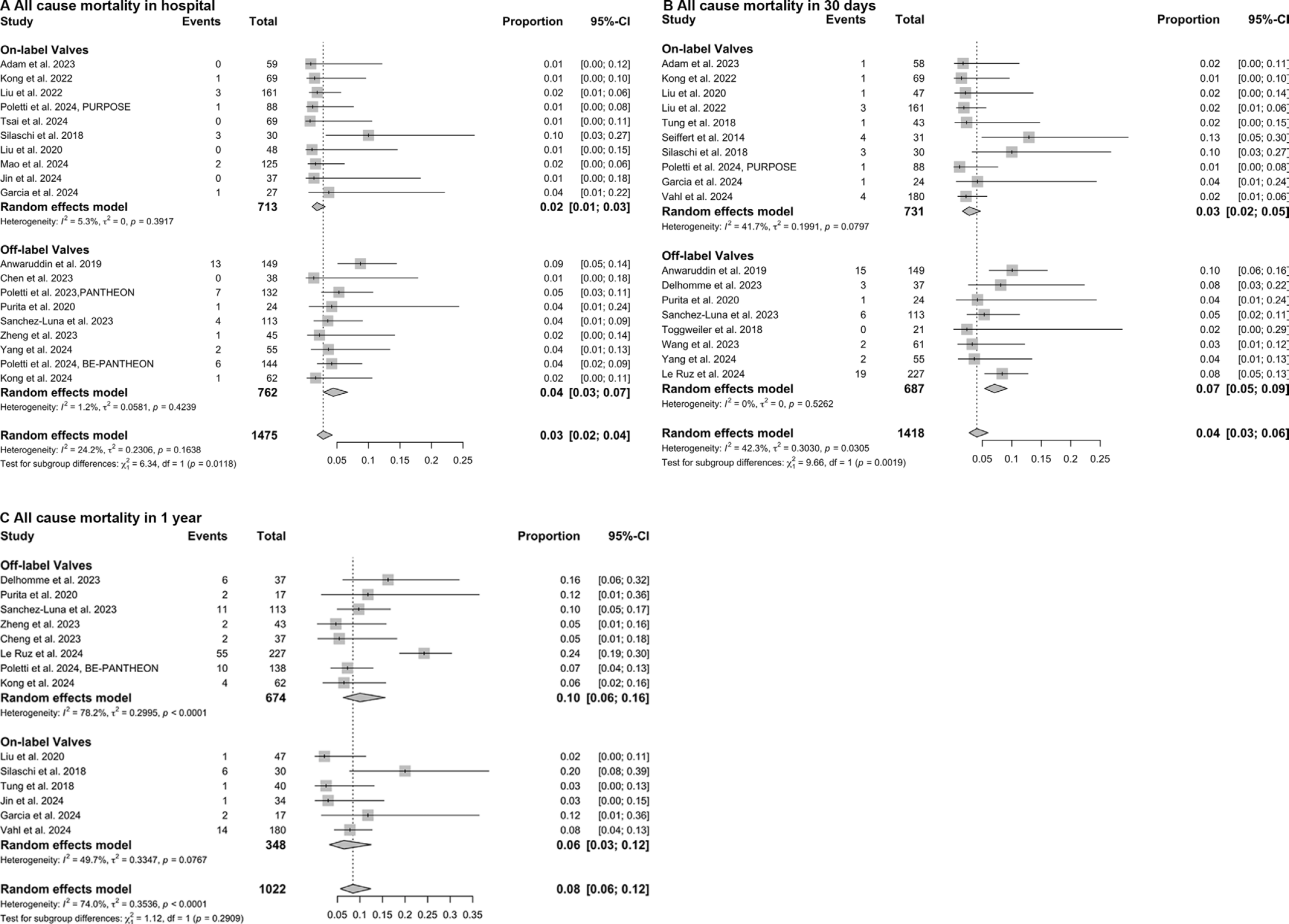
Forest plots of all-cause mortality in on-label and off-label transcatheter aortic valve replacement for aortic regurgitation. The analysis included 19 studies with 1475 patients for in-hospital mortality, 18 studies with 1418 patients for 30-day mortality, and 14 studies with 1022 patients for 1-year mortality. Pooled estimates were generated using the DerSimonian-Laird random-effects mode random-effects model with logit transformation. Subgroup definitions were: on-label devices and off-label valves. Pairwise comparisons were adjusted for multiple testing using the false discovery rate method.

To enhance statistical power, we conducted an additional comparison between on-label and off-label devices. The results are illustrated in figure 4. In-hospital mortality was 2% (95% CI: 1% to 3%) in the on-label group and 4% (95% CI: 3% to 7%) in the off-label group, with a significant subgroup difference (χ^2^=6.34, p=0.012). At 30 days, mortality remained lower in the on-label group (3% (95% CI: 2% to 5%)) compared with the off-label group (7% (95% CI: 5% to 9%)), with a statistically significant subgroup difference (χ^2^=9.66, p=0.002). At the 1-year follow-up, mortality was 6% (95% CI: 3% to 12%) for on-label devices and 10% (95% CI: 6% to 15%) for off-label devices, but the difference was not statistically significant (χ^2^=1.12, p=0.291).

### Sensitivity analyses

The results of SA were generally in line with the main findings, supporting the robustness of our conclusions. Notably, transfemoral on-label implantation was associated with lower in-hospital and 30-day mortality versus off-label:SE, and exclusion of European studies accentuated this difference. For device success, subgroup differences in the primary analysis did not translate into significant pairwise differences after FDR correction. In SA, however, subgroup differences persisted in most scenarios, with on-label devices showing significantly higher success than off-label SE, but these differences disappeared when Asian studies were excluded, reflecting the predominance of off-label SE series with lower success rates in Asia. Detailed results are provided in online supplemental table 6.

### Meta-regression analysis for 1-year all-cause mortality

For the stepwise multivariate meta-regression, 14 covariates with 10 corresponding estimates of 1-year all-cause mortality were analysed (table 3). In univariate meta-regression, coronary heart disease (coefficient: 0.567, p=0.001) and atrial fibrillation (coefficient: 0.466, p=0.010) were significantly associated with increased 1-year all-cause mortality (online supplemental figure 1).

**Table 3:** Meta-regression analysis for 1-year all-cause mortality

Variable	No. of studies	Univariate			Multivariate	
		**Coefficient (95% CI**)	P **value**	**Figure**	**Coefficient (95% CI**)	P **value**
Age	10	0.005 (−0.018 to 0.029)	0.677			
Female	16	0.291 (−0.145 to 0.728)	0.212			
HT	16	−0.042 (−0.422 to 0.337)	0.830			
DM	16	−0.185 (−0.841 to 0.472)	0.590			
CHD	12	0.567 (0.315 to 0.818)	0.001	S1	0.45 (0.118 to 0.782)	0.026
AF	16	0.466 (0.161 to 0.771)	0.010	S2	0.081 (−0.229 to 0.39)	0.621
COPD	10	0.183 (−0.189 to 0.555)	0.364			
PAD	14	0.032 (−0.397 to 0.461)	0.886			
NYHA III/IV	15	0.163 (−0.2 to 0.525)	0.395			
Bicuspid	10	−0.101 (−0.566 to 0.365)	0.683			
Mean LVEF	10	−0.01 (−0.02 to, to 0.001)	0.067			
Access*	13	0.021 (−0.031 to 0.074)	0.444			
THV type†	14	−0.028 (−0.089 to 0.032)	0.383			
Annulus perimeter	10	−0.007 (−0.019 to 0.005)	0.267			

* Access: binary variable, transfemoral or transapical approach.

† HV type: ternary variable, on-label, off-label self-expanding valves or off-label balloon-expandable valves.

AF, atrial fibrillation; CHD, coronary heart disease; COPD, chronic obstructive pulmonary disease; DM, diabetes mellitus; HT, hypertension; LVEF, left ventricular ejection fraction; NYHA, New York Heart Association; PAD, peripheral arterial disease; THV, transcatheter heart valve.

After adjusting for multiple variables in the multivariate model, CHD remained significant (coefficient: 0.45, 95% CI: 0.118 to 0.782, p=0.026), confirming it as an independent predictor of 1-year mortality. In contrast, AF lost statistical significance (coefficient: 0.081, 95% CI: −0.229 to 0.39, p=0.621), suggesting that its effect may be confounded by other clinical factors.

Age, mean LVEF, access route, THV type and annulus perimeter were not significantly associated with mortality in either univariate or multivariate analyses (p>0.05 for all). These findings indicate that while CHD is a key determinant of 1-year mortality, other patient comorbidities did not show an independent effect after accounting for confounding factors.

## Discussion

This meta-analysis found that the overall 1-year all-cause mortality after TAVR for AR was 10.4%. Among valve types, on-label devices had the lowest mortality, followed by off-label: BE devices, with off-label:SE devices exhibiting the highest mortality. However, these differences did not reach statistical significance.

Subgroup analysis demonstrated superior procedural success for on-label devices, with significantly lower rates of moderate or severe AR and valve migration compared with off-label devices. Among off-label valves, SE valves were associated with a higher incidence of valve migration and SVI than BE valves, suggesting challenges in achieving stable anchoring.

Meta-regression analysis identified CHD as an independent predictor of 1-year mortality (p=0.026), while age, mean LVEF, access route, THV type and annulus perimeter were not significantly associated with mortality. These findings highlight the impact of patient comorbidities on long-term survival and emphasise the need for further research to refine patient selection and procedural strategies. In this context, this finding underscores the importance of comprehensive coronary assessment before TAVR in AR patients. In the presence of significant coronary artery disease, procedural planning may need to incorporate strategies such as concomitant percutaneous coronary intervention or staged revascularisation, while ensuring that valve selection and implantation technique maintain future coronary access. Furthermore, the presence of CHD warrants intensified secondary prevention measures, optimisation of medical therapy, and closer longitudinal follow-up to mitigate the risk of adverse cardiovascular events after TAVR. These considerations highlight that comorbid CHD not only impacts prognosis but also shapes both procedural and long-term management strategies.

In the two-group analysis (on-label vs off-label), both in-hospital and 30-day mortality were significantly lower in the on-label group, whereas 1-year mortality showed no statistical difference. In contrast, in the three-group analysis (on-label, off-label:SE, off-label:BE), none of the mortality outcomes—including in-hospital, 30-day and 1-year mortality—differed significantly among the groups. This discrepancy may be attributed to the reduced statistical power caused by subdividing the off-label group, as well as the relatively smaller sample size in each subgroup. The lack of a significant mortality difference in the three-group analysis suggests that while procedural outcomes differ among valve types, these advantages may not necessarily translate into mid-term survival benefits. Furthermore, the absence of statistical significance in 1-year mortality comparisons across both analyses indicates that mid-term outcomes may be driven more by patient-specific factors rather than valve selection alone.

Our findings are consistent with the study by Liu *et al*,^16^ who conducted a meta-analysis of 31 studies (1851 patients) comparing 30-day mortality between on-label and off-label devices. Their results showed a significantly lower mortality rate in the on-label group (2.6%) compared with off-label (5.1%, p=0.006), aligning with our two-group analysis. However, their study included small-sample studies with as few as 10–20 patients, which may have introduced variability due to early procedural experiences and less mature techniques, leading to poorer reported outcomes. Additionally, overlapping patient populations across multiple studies may have inflated the weight of certain cohorts, affecting the accuracy of their estimates. In contrast, our study excluded very small-sample studies, ensured non-overlapping patient inclusion and implemented stricter selection criteria, yielding a more robust and generalisable mortality estimate.

Conversely, our results diverge from Samimi *et al*,^17^ who reported a substantially higher 1-year mortality in the off-label group (24%) compared with the on-label group (6%) (p<0.01). This discrepancy is likely due to differences in study selection criteria. Notably, Samimi *et al* included first-generation devices (eg, Testa 2014, Munoz Garcia 2017), which have lower procedural success rates and higher complications, contributing to increased mortality. Furthermore, their study included small-sample studies (<20 patients, eg, Paraggio 2022, Purita 2020), introducing greater variability and potential bias. Additionally, some studies (eg, Le Ruz 2024) may have misclassified follow-up patients as deceased, potentially leading to overestimation of mortality in the off-label group.

By excluding first-generation devices, minimising selection bias and applying rigorous methodological criteria, our study provides a more precise assessment of mortality outcomes in new-generation TAVR for AR. These refinements strengthen the reliability of our findings and provide a clearer understanding of the comparative effectiveness of different valve types.

Although the subgroup analysis revealed no statistically significant difference in 1-year all-cause mortality among the on-label, off-label:SE and off-label:BE groups, notable differences in procedural outcomes suggest potential long-term prognostic implications. The on-label group demonstrated the highest procedural success rate, with significantly lower rates of valve migration and moderate or severe AR compared with off-label devices. Given that post-TAVR moderate or severe AR is associated with poorer long-term survival, these findings underscore the procedural advantages of on-label devices, which may contribute to better long-term outcomes.^18 19^ Additionally, patients treated with on-label devices had lower rates of NYHA class III–IV symptoms and PPI, further illustrating their haemodynamic stability and clinical benefit. Collectively, these results highlight that on-label devices provide superior procedural safety and post-TAVR haemodynamic performance, making them a preferred option when applicable.

For off-label devices, patient selection remains crucial to optimising outcomes. Notably, off-label:BE devices exhibited a lower rate of SVI and higher procedural success than off-label:SE devices, despite being primarily anchored at the annulus. In contrast, off-label:SE devices demonstrated a higher incidence of valve migration, suggesting that achieving stable anchoring remains a challenge. These findings indicate that anatomical suitability, particularly annular characteristics, should be carefully evaluated to optimise stability and procedural success. From a clinical perspective, the higher rate of SVI with off-label:SE devices is also significant, as it is associated with prolonged procedure duration and increased risks of vascular injury and conduction disturbances. Although the deployment of a second valve can usually resolve severe paravalvular regurgitation and restore haemodynamic function, its necessity highlights the procedural difficulties of self-expanding devices in non-calcified AR and reinforces the importance of judicious device selection to minimise this complication.

However, it should be noted that heterogeneity was observed within the off-label subgroups, particularly for device success. This variability may stem from differences in patient selection criteria, geographic practice patterns, anatomical characteristics (eg, annulus size, leaflet morphology) and operator experience across the included studies. Our SA support this interpretation. In most scenarios, subgroup differences in device success persisted, with on-label devices consistently outperforming off-label:SE. However, these differences disappeared when Asian studies were excluded, likely due to the predominance of Asian cohorts using off-label:SE devices, which reported lower success rates. Similarly, the observation that transfemoral on-label implantation was associated with lower early mortality compared with off-label:SE indicates that access route may represent another important effect modifier. Taken together, such heterogeneity reinforces the need for cautious interpretation of subgroup results and supports the development of standardised selection criteria to improve outcomes in off-label TAVR for AR.

To further evaluate the role of TAVR in the treatment of AR, we reviewed four studies comparing TAVR and SAVR published after 2016, when newer-generation TAVR devices became widely available.^20^,^23^ Our pooled analysis demonstrated that TAVR was associated with a lower in-hospital mortality rate compared with SAVR (OR: 0.71, 95% CI: 0.58 to 0.89). Additionally, the risk of in-hospital stroke (OR: 0.49, 95% CI: 0.32 to 0.77), AKI (OR: 0.50, 95% CI: 0.45 to 0.56) and blood transfusion requirements (OR: 0.19, 95% CI: 0.14 to 0.26) were significantly lower in the TAVR group (online supplemental figure 2 and table 7). These findings highlight the procedural advantages of TAVR, particularly in reducing perioperative complications and facilitating a less invasive approach in high-risk patients. However, TAVR was associated with a significantly higher risk of PPI compared with SAVR (OR: 1.78, 95% CI: 1.57 to 2.03). This increased risk of conduction disturbances may be related to the positioning of transcatheter valves relative to the conduction system, particularly in self-expanding valves. While newer-generation TAVR devices have improved in minimising conduction disturbances, PPI rates remain an important consideration when selecting candidates for TAVR, particularly in younger patients with longer life expectancy. It should be emphasised that the comparison between TAVR and SAVR represents a post hoc exploratory analysis rather than a predefined study objective, and results should therefore be interpreted with caution given differences in patient selection, risk profile and valve types.

Overall, our findings suggest that TAVR offers a less invasive alternative to SAVR with lower procedural risks and comparable in-hospital survival in AR patients. However, the higher incidence of conduction disturbances highlights the need for further advancements in valve design and patient selection strategies. Additionally, as most of the included studies focused on high-risk patients, the role of TAVR in intermediate-risk and low-risk AR populations remains uncertain. Future RCTs directly comparing new-generation TAVR versus SAVR in AR-specific populations are warranted to refine patient selection and optimise long-term outcomes.

## Study limitations

This study has several limitations. First, despite including a large number of studies, the sample size of individual studies remained relatively small, limiting statistical power and the generalisability of the findings. Second, most included studies were retrospective observational studies, inherently prone to selection bias and residual confounding. Third, long-term outcomes beyond 1 year remain uncertain, as the majority of studies focused on in-hospital and short-term follow-up data. The durability of newer generation transcatheter valves in AR patients, as well as their long-term impact on functional status, structural valve deterioration and reintervention rates, warrants further investigation. Additionally, heterogeneity in patient selection, procedural techniques and device types may have contributed to variations in reported outcomes.

Finally, potential publication bias was detected in certain outcomes, as indicated by the Egger regression test, suggesting that selective publication may have influenced effect size estimates (online supplemental figure 3). Although rigorous inclusion criteria were applied to minimise bias, its presence cannot be entirely ruled out. Future prospective, large-scale studies with extended follow-up are needed to validate these findings and further refine the role of different TAVR devices in AR management.

## Conclusions

This meta-analysis demonstrated that newer-generation TAVR devices achieve high procedural success rates in AR patients, with no significant difference in 1-year mortality among on-label, off-label:SE and off-label:BE devices. However, on-label devices exhibited superior procedural outcomes, including lower rates of valve migration and moderate or severe AR, reinforcing their procedural advantages. While TAVR remains a viable treatment option for AR, further long-term studies are needed to assess valve durability and its impact on clinical outcomes over time.

## Supplementary material

10.1136/openhrt-2025-003482 online supplemental file 1

## Data Availability

Data sharing not applicable as no datasets generated and/or analysed for this study.
