# Supplementary material for: Comparative outcomes of on-label and off-label transcatheter aortic valve replacement for aortic regurgitation: a systematic review and meta-analysis
Source: Open Heart. 2025 Oct 17;12(2):e003482. doi: 10.1136/openhrt-2025-003482 (PMC12542711; doi:10.1136/openhrt-2025-003482)
Supplement: online supplemental file 1 [file openhrt-12-2-s001.docx]

Supplementary Table 1 Classification and Key Features of TAVR Devices in Aortic Regurgitation

| Device Category | Representative Devices | Anchoring Mechanism | Deployment Method | Regulatory Status for AR |
| --- | --- | --- | --- | --- |
| **On-label** | JenaValve, J-Valve | Dual fixation: leaflet graspers + annular anchoring | Self-expanding | Specifically approved for AR |
| **Off-label:BE** | Sapien 3, Myval | Annular anchoring via balloon inflation | Balloon-expandable | Approved for AS, used off-label in AR |
| **Off-label:SE** | Evolut PRO, ACURATE neo, VenusA-Valve | Gradual self-expansion, flared structure anchoring at annulus and/or sinotubular junction | Self-expanding | Approved for AS, used off-label in AR |

Supplemental Table 2 Search Strategy for Meta-Analysis on Aortic Valve Regurgitation

| **PubMed** **Search Strategy** | | | |
| --- | --- | --- | --- |
| **#1** | **'("Aortic Valve Insufficiency"[MeSH Terms] OR "aortic regurgitation" OR "aortic valve regurgitation" OR "aortic valve insufficiency") AND ("Transcatheter Aortic Valve Replacement"[MeSH Terms] OR "transcatheter aortic valve implantation" OR "transcatheter aortic valve replacement" OR "TAVR" OR "TAVI" OR "percutaneous aortic valve replacement" OR "transfemoral aortic valve implantation" OR "transapical aortic valve implantation")'** | | |
| **Embase Search Strategy** | | | |
| **#1** | ('aortic valve insufficiency'/exp OR 'aortic regurgitation' OR 'aortic valve insufficiency') AND ('transcatheter aortic valve replacement'/exp OR 'transcatheter aortic valve implantation' OR TAVR OR TAVI) | | |
| **Cochrane Search Strategy** | | |  |
| #1 | | (aortic regurgitation)：ti，ab，kw (Word variations have been searched) |  |
| #2 | | (aortic valve insufficiency)：ti，ab，kw (Word variations have been searched) |  |
| #3 | | MeSH descriptor： [Aortic Valve Insufficiency] explode all trees |  |
| #4 | | #1 OR #2 OR #3 |  |
| #5 | | (transcatheter aortic valve replacement)：ti，ab，kw (Word variations have been searched) |  |
| #6 | | (transcatheter aortic valve implantation)：ti，ab，kw (Word variations have been searched) |  |
| #7 | | (TAVR)：ti，ab，kw (Word variations have been searched) |  |
| #8 | | (TAVI)：ti，ab，kw (Word variations have been searched) |  |
| #9 | | MeSH descriptor： [Transcatheter Aortic Valve Replacement] explode all trees |  |
| #10 | | #5 OR #6 OR #7 OR #8 OR #9 |  |
| #11 | | #4 AND #10 |  |
| **Web of Science Search Strategy** | | |  |
| **#1** | | TS=("aortic regurgitation" OR "aortic valve insufficiency") AND TS=("transcatheter aortic valve replacement" OR "transcatheter aortic valve implantation" OR "TAVR" OR "TAVI") |  |

TAVI: transcatheter aortic valve implantation; TAVR: transcatheter aortic valve replacement

Supplemental Table 3 Methodological characteristics of included studies

| **Author** | **Design** | **Study Period** | **Region** | **VARC** |
| --- | --- | --- | --- | --- |
| **Kong et al. 2022** | Retrospective, single-center study | September 2016 to September 2021 | China | VARC-2 |
| **Chen et al. 2022** | Retrospective, single-center study | October 2018 to December 2020 | China | VARC-2 |
| **Tung et al. 2018** | Prospective, multicenter study | April 2018 to June 2018 | China | VARC-2 |
| **Wang et al. 2022** | Retrospective, multicenter study | January 2019 to December 2021 | China | VARC-3 |
| **Chen et al. 2023** | Retrospective-prospective, single-center study | January 2019 to September 2022 | China | - |
| **Sanchez-Luna et al. 2023** | Retrospective, multicenter study | July 2020 to June 2021 | Spain, Italy, Colombia, VARC-3  Kazakhstan, Argentina​​ | |
| **Yang et al.2024** | Retrospective single-center study | September 2021 to September 2023 | China | - |
| **Yu et al.2024** | Retrospective, single-center study | April 2019 to January 2023 | China | VARC-3 |
| **Liu et al. 2022** | Retrospective study, single-center study | March 2014 to July 2019 | China | VARC-2 |
| **Seiffert et al. 2014** | Retrospective, multicenter study | April 2012 to October 2013 | Germany | VARC-2 |
| **Le Ruz et al.2024** | Prospective, multicenter study | 2015 to 2021 | France | VARC-3 |
| **Poletti et al. 2024, BE-PANTHEON** | Retrospective, multicenter study | February 2018 to July 2023 | Europe and USA | VARC-3 |
| **Poletti et al. 2024, PURPOSE** | Retrospective, multicenter study | April 2015 to September 2023 | Europe and USA | VARC-3 |
| **Tsai et al. 2024** | Retrospective, multicenter study | September 2021 and May 2023 | Germany, Ireland, UK​, China | VARC-3 |
| **Sawaya et al. 2017** | Prospective, multicenter study | July 2007 and September 2016 | Europe | VARC-2 |
| **Anwaruddin et al. 2019** | Registry study analysis | January 2014 and December 2017 | USA | VARC-2 |

VARC-1: Valve Academic Research Consortium-1; VARC-2: Valve Academic Research Consortium-2; VARC-3: Valve Academic Research Consortium-3

Supplemental Table 3(continued)

| **Author** | **Design** | **Study Period** | **Region** | **VARC** |
| --- | --- | --- | --- | --- |
| **Poletti et al. 2023, PANTHEON** | Retrospective, multicenter study | May 2014 and September 2022 | Europe and USA | VARC-3 |
| **Yoon et al. 2017** | Retrospective and prospective multicenter study | Initiated in August 2016 | Europe, North America, VARC-2  and Asia-Pacific | |
| **Hinkov et al. 2024** | Retrospective, single-center study | Initiated in January 2019 | Germany | VARC-3 |
| **Garcia et al. 2023** | Retrospective, multicenter study | 2018 to 2022 | the North American VARC-3 | |
| **Purita et al. 2020** | Retrospective, multicenter study | September 2016 and October 2018 | Europe | VARC-2 |
| **Toggweiler et al. 2018** | Retrospective, multicenter study | May 2015 to July 2017 | Europe and Israel - | |
| **Zheng et al. 2023** | Retrospective study, single-center | July 2020 and June 2021 | China | VARC-2 |
| **De Backer et al. 2018** | Prospective, multicenter study​ | 2007 to 2017 | Europe, North America, - Middle East, Asia | |
| **Vahl et al.2024** | Prospective, multicenter study​ | June 2018 to August 2022 | USA | VARC-2 |
| **Silaschi et al. 2018** | Prospective, multicenter study | 2012 to 2015 | Germany and UK | VARC-1 |
| **Liu et al. 2020** | Retrospective, single-center study​ | May 2014 through October 2018 | China | VARC-2 |
| **Mao et al. 2024** | Prospective, multicenter study​ | January 2018 to May 2021 | China | VARC-2 |
| **Jin et al. 2024** | Retrospective, single-center study​ | January 2018 to December 2022 | China | - |
| **Delhomme et al. 2023** | Retrospective and prospective multicenter study​ | January 2015 to July 2021 | France | VARC-2 |
| **Adam et al. 2023** | Prospective, multicenter study​ | September 2021 and July 2022 | Germany | VARC-3 |
| **Kong et al. 2024** | Retrospective, single-center study | September 2019 to February 2022 | China | VARC-3 |

VARC-1: Valve Academic Research Consortium-1; VARC-2: Valve Academic Research Consortium-2; VARC-3: Valve Academic Research Consortium-3

Supplemental Table 4 Comorbidities of included studies

|  | **HT(%)** | **DM(%)** | **CAD(%)** | **AF(%)** | **CODP(%)** | **CKD(%)** | **PAD(%)** | **Previous CABG (%)** | **Previous PCI (%)** | **Previous MI(%)** | **Previous Stroke (%)** | **Previous NYHA III/IV (%)** |
| --- | --- | --- | --- | --- | --- | --- | --- | --- | --- | --- | --- | --- |
| **Kong et al. 2022** | 69.6 | 13 | 27.5 | 26.1 | 20.3 | - | 10.1 | 1.4 | 5.8 | 0 | - | 76.8 |
| **Chen et al. 2022** | 77 | 14 | 38 | - | 29 | 13 | 18 | - | - | 22 | - | 68 |
| **Tung et al. 2018** | 74.4 | 9.3 | 11.6 | 23.3 | 58.1 | - | 46.5 | 2.3 | 4.7 | - | 9.3 | 97.7 |
| **Wang et al. 2022** | 67.2 | 14.8 | 29.5 | 29.5 | - | - | - | - | - | - | - | 82 |
| **Chen et al. 2023** | 62.2 | 18.9 | 37.8 | 35.1 | 8.1 | - | 5.4 | 5.4 | 8.1 | - | 5.4 | - |
| **Sanchez-Luna et al. 2023** | 85.8 | 26.5 | - | 31 | 22.1 | 15.9 | 9.7 | 3.5 | 5.3 | 5.3 | 7.1 | 62.8 |
| **Yang et al.2024** | 65.5 | 29.1 | 14.5 | - | 1.8 | 3.6 | - | - | - | - | - | 81.8 |
| **Yu et al.2024** | - | - | - | - | - | - | - | - | - | - | - | 53.4 |
| **Liu et al. 2022** | 66.5 | 14.9 | 32.3 | 22.4 | 31.1 | - | - | - | 2.5 | - | - | 55.8 |
| **Seiffert et al. 2014** | 83.9 | 12.9 | 64.5 | 19.3 | 29 | - | 19.3 | 22.6 | 32.2 | 35.5 | 22.6 | 38.7 |
| **Le Ruz et al.2024** | 64.8 | 16.5 | 38.2 | 46.3 | - | 51.6 | 19.1 | 12.4 | 25.8 | - | 15.1 | 77.3 |
| **Poletti et al. 2024 BE-PANTHEON** | 85 | 24.3 | 22.9 | 34 | 20.8 | 15.3 | 11.8 | 9 | 11.8 | 11.1 | 4.9 | 69.4 |
| **Poletti et al. 2024 PURPOSE** | 84 | 18.2 | - | 45.5 | 17 | 19.3 | 11.4 | 15.9 | 11.4 | 5.7 | 13.6 | 77 |
| **Tsai et al. 2024** | 89.7 | 22.1 | 45.6 | 45.6 | 14.7 | - | 11.8 | 16.2 | 11.8 | - | 13.2 | 75 |
| **Sawaya et al. 2017** | 84 | 24 | - | 24 | 29 | 11 | 34 | 24 | 34 | 26 | - | - |
| **Anwaruddin et al. 2019** | 78.7 | 19.5 | 4900 | - | - | 13 | 29 | 27 | - | - | 13 | 91 |
| **Kong et al. 2024** | 66.1 | 16.1 | - | 38.7 | 14.5 | 6.5 | 11.3 | 3.2 | 8.1 | 3.2 | - | 61.3 |
| **Silaschi et al. 2018** | 80 | 16.7 | 46.7 | 30 | 16.7 | 36.7 | 10 | 16.7 | 26.7 | 3.3 | 3.3 | 90 |
| **Liu et al. 2020** | 66 | 8.5 | 23.4 | 19.1 | - | - | 21.3 | 4.3 | - | 0 | 6.4 | 74.5 |

§ Dialysis; HT, Hypertension; DM, Diabetes Mellitus; CHD, Coronary Heart Disease; AF, Atrial Fibrillation; COPD, Chronic Obstructive Pulmonary Disease; CKD, Chronic Kidney Disease; PAD, Peripheral Arterial Disease; MI, Myocardial Infarction; NYHA, New York Heart Association

Supplemental Table 4(continued)

|  | **HT(%)** | **DM(%)** | **CHD(%)** | **AF(%)** | **CODP(%)** | **CKD(%)** | **PAD(%)** | **Previous CABG (%)** | **Previous PCI (%)** | **Previous MI(%)** | **Previous Stroke (%)** | **Previous NYHA III/IV (%)** |
| --- | --- | --- | --- | --- | --- | --- | --- | --- | --- | --- | --- | --- |
| **Mao et al. 2024** | 23.2 | 19.2 | 23.2 | 23.2 | 8.8 | 23.2**§** | 54.4 | 23.2 | 4 | - | 2.4 | 98.4 |
| **Jin et al. 2024** | 58.3 | 5.6 | 19.4 | 11.1 | - | 16.7 | 58.3 | - | - | 2.8 | 44.4 | 88.9 |
| **Delhomme et al. 2023** | 67.6 | 21.6 | 40.5 | 40.5 | 16.2 | 2.7**§** | - | 13.5 | - | - | - | 83.8 |
| **Matti Adam et al. 2023** | 91.4 | 24.1 | 43.1 | 58.6 | 15.5 | - | 12.1 | 22.4 | 29.3 | 8.6 | 13.8 | 74.1 |
| **Poletti et al. 2023 PANTHEON** | 76 | 20 | - | 43.2 | 15.9 | - | 17.4 | 2650 | 21.2 | 13.6 | 9.1 | 78 |
| **Yoon et al. 2017** | 78.8 | 10.4 | 49.1 | 37.3 | 33 | - | 21.2 | 13.7 | 28.8 | 23.1 | 11.8 | 87.7 |
| **Hinkov et al. 2024** | - | - | - | - | - | - | - | - | - | - | - | - |
| **Garcia et al. 2023** | 89 | 19 | - | 33 | 26 | - | 15 | 15 | 48 | 15 | 15 | 96 |
| **Purita et al. 2020** | 75 | 16.6 | 25 | 4.1 | - | 4.1**§** | - | 4.1 | 4.1 | 0 | - | 95.8 |
| **Toggweiler et al. 2018** | - | 10 | - | 40 | - | - | - | 30 | - | - | - | 85 |
| **Zheng et al. 2023** | 48.9 | 26.7 | 15.6 | 8.9 | - | 22.2 | 11.1 | 0 | 11.1 | 0 | - | 95.6 |
| **De Backer et al. 2018** | 80 | 15 | - | 34 | - | 65 | 27 | 14 | 24 | 26 | - | - |
| **Vahl et al.2024** | 83 | 14 | - | 40 | 18 | 33 | 12 | 12 | 23 | - | 11 | 68 |

§ Dialysis; HT, Hypertension; DM, Diabetes Mellitus; CHD, Coronary Heart Disease; AF, Atrial Fibrillation; COPD, Chronic Obstructive Pulmonary Disease; CKD, Chronic Kidney Disease; PAD, Peripheral Arterial Disease; MI, Myocardial Infarction; NYHA, New York Heart Association

Supplemental Table 5 Echocardiographic and structural characteristics of included studies

|  | **Bicuspid (%)** | **Mean LVEF (%)** | **Mean LVEDD (mm)** | **﻿Annulus ﻿Perimeter mm** |
| --- | --- | --- | --- | --- |
| **Kong et al. 2022** | - | 50.84 ± 12.38 | - | - |
| **Chen et al. 2022** | - | ﻿43.9 ± 13.9 | ﻿59.2 ± 10.2 | ﻿80.9 ± 8.6 |
| **Tung et al. 2018** | 4.7 | 55.9 ± 10.8 | - | - |
| **Wang et al. 2022** | 3.3 | 54.3 ± 12.4 | 58.0 ± 6.3 | ﻿78.5 ± 7.1 |
| **Chen et al. 2023** | 5.4 | 53.1 ± 12.1 | 62.5 ± 7.3 | 81.7 ± 6.8 |
| **Sanchez-Luna et al. 2023** | 7.1 | 43.4 ± 14.7 | 63.6 ± 9.2 | 88.5 ± 8.0 |
| **Yang et al.2024** | 9.1 | 55.2 ± 11.1 | 57.4 ± 7.4 | 81.0 ± 7.5 |
| **Yu et al.2024** | 6.7 | 53.2 ± 14.4 | - | 81.0 ± 8.2 |
| **Liu et al. 2022** | 8.1 | 52.3 ± 12.8 | 65.1 ± 9.3 | - |
| **Seiffert et al. 2014** | 0 | - | - | 24.7 (20.8–27.0) |
| **Le Ruz et al.2024** | - | 46.0(35.0-60.0) | - | - |
| **Poletti et al. 2024 BE-PANTHEON** | 9 | 41.0(30.0–60.0) | 63.0 (58.0–70.0) | - |
| **Poletti et al. 2024 PURPOSE** | 0 | 50.0(40.0–55.0) | 60.0 (54.0–63.0) | 77.0 (74.0–81.0) |
| **Tsai et al. 2024** | 0 | 49.0(40.0–55.0) | 56.0 (7.1–62.0) | 82.6 (76.2–86.2) |
| **Sawaya et al. 2017** | - | 41.3 ± 14.0 | 58.4 ± 10.5 | - |
| **Anwaruddin et al. 2019** | - | 42.0 ± 17.5 | - | - |
| **Kong et al. 2024** | - | 54.1 ± 10.8% | 61.6 ± 5.6 | 78.6 ± 7.0 |
| **Silaschi et al. 2018** | - | 49.6 ± 13.3% | - | 76.3 ± 6.0 |
| **Liu et al. 2020** | 6.4 | 52.3 ± 12.4 | 59.2 ± 8.4 | 85.1 ± 6.9 |
| **Mao et al. 2024** | 6.4 | 49.9 ± 7.6 | - | 93.9 ± 2.5 |

Supplemental Table 5(continued)

|  | **Bicuspid (%)** | **Mean LVEF (%)** | **Mean LVEDD (mm)** | **﻿Annulus ﻿Perimeter mm** |
| --- | --- | --- | --- | --- |
| **Jin et al. 2024** | - | 53.5 (39.75–58.00) | - | - |
| **Delhomme et al. 2023** | - | 45 (40–56) | - | 85 (78–88.1) |
| **Matti Adam et al. 2023** | - | >50%: 34 (59%), 41%-50%: 22 (38%), 31%-40%: 7 (12%), <31%: 9 (16%) | 58.3 ± 7.5 | 80.3 ± 9.7 |
| **Poletti et al. 2023 PANTHEON** | 5 | 45 ± 13 | 58.9 ± 9.3 | 78 (74-83) |
| **Yoon et al. 2017** | - | 44.3 ± 14.5% | - | 79.2 ± 11.5 |
| **Hinkov et al. 2024** | - | - | - | - |
| **Garcia et al. 2023** | 4 | 54(37-60) | 55 ± 9 | 81 ± 10.5 |
| **Purita et al. 2020** | 4.1 | 48.5 ( 30–65) ¢ | 60 ( 41–83) ¢ | 77.1 (62.6–81.3)¢ |
| **Toggweiler et al. 2018** | - | 48 ± 14 | 58 ± 7 | 75 ± 6 |
| **Zheng et al. 2023** | 2.2 | ﻿41.5 ± 4.6 | ﻿62.1 ±4.9 | 82.6 ± 7.9 |
| **De Backer et al. 2018** | - | 45 ± 15 | 60 ± 11 | - |
| **Vahl et al.2024** | 0 | 53.8 ± 11.4 | 39.6 ± 10.2 | 79.1 ± 6.1 |

¢ median(min-max). LVEF, Left Ventricular Ejection Fraction; LVEDD: Left Ventricular End-Diastolic Diameter

Supplemental Table 6 Sensitivity Analysis

|  |  |  | **On-label devices** | **Off-label SE** | **Off-label BE** |  |  |
| --- | --- | --- | --- | --- | --- | --- | --- |
| **Outcomes** | **n** | **N** | **Event rate (95% CI)** | **Event rate (95% CI)** | **Event rate (95% CI)** | **χ^2^** | ***P**** |
|  | | | **SA-1** | | |  | |
| all cause mortality in 1 year | 12 | 758 | 7.6% (3.8–14.6%) | 6.9% (3.5–13.2%) | 9.9% (6.4–14.9%) | 0.98 | 0.614 |
| all cause mortality in 30 days | 14 | 860 | 3.6% (1.8–6.9%) | 3.6% (1.5–8.3%) | 6.1% (3.2–11.3%) | 1.59 | 0.452 |
| all cause mortality in hospital | 16 | 1159 | 2.8% (1.5–5.4%) | 4.1% (2.3–7.1%) | 3.9% (2.1–7.1%) | 0.82 | 0.663 |
| SVI | 20 | 1352 | 3.1% (1.7–5.6%) | 18.0% (13.1–24.2%) | 5.4% (3.3–8.8%) | 34.18 | 0.000¡£ |
| Moderate or severe AR in hospital | 23 | 1451 | 2.7% (1.6–4.6%) | 5.0% (3.1–8.1%) | 9.2% (6.2–13.6%) | 13.5 | 0.001¢ |
| Device success | 15 | 985 | 94.9% (91.0–97.2%) | 79.8% (73.7–84.8%) | 89.4% (67.8–97.1%) | 19.02 | 0.000¡ |
|  | | | **SA-2** | | |  | |
| all cause mortality in 1 year | 7 | 447 | - | 6.5% (3.6–11.8%) | 9.9% (6.4–14.9%) | 1.22 | 0.270 |
| all cause mortality in 30 days | 10 | 785 | 1.9% (0.9–4.2%) | 7.6% (4.9–11.6%) | 6.1% (3.2–11.3%) | 9.20 | 0.010¡¢ |
| all cause mortality in hospital | 12 | 975 | 1.2% (0.4–4.2%) | 6.2% (4.3–8.9%) | 3.9% (2.1–7.1%) | 7.15 | 0.028¡ |
| SVI | 18 | 1261 | 1.8% (0.9–3.6%) | 17.9% (13.5–23.4%) | 5.4% (3.3–8.8%) | 45.7 | 0.000¡¢£ |
| Moderate or severe AR in hospital | 20 | 1438 | 2.2% (1.0–4.9%) | 5.9% (3.8–8.9%) | 9.2% (6.2–13.6%) | 10.27 | 0.006¡¢ |
| Device success | 15 | 1090 | 96.3% (91.3–98.4%) | 81.6% (75.4–86.6%) | 89.4% (67.8–97.1%) | 12.91 | 0.002¡ |
|  | | | **SA-3 excluding Asian cohorts** | | |  | |
| all cause mortality in 1 year | 6 | 515 | 11.7% (5.9–21.9%) | - | 9.9% (6.4–14.9%) | 0.17 | 0.678 |
| all cause mortality in 30 days | 11 | 754 | 4.3% (1.8–9.8%) | 9.4% (6.0–14.6%) | 6.1% (3.2–11.3%) | 3.11 | 0.211 |
| n:Number of Studies; N:Overall Sample Size; ¡ On-label devices vs Off-label SE; ¢ On-label devices vs Off-label BE; £ Off-label SE vs Off-label BE;  * Test for subgroup differences;SA-1, excluding studies with mean STS > 8%; SA-2, studies in which ≥90% of patients underwent transfemoral access | | | | | | | |
|  | | | | | | | |
| Supplemental Table 6 (continued) | | | | | | | |
|  | | | **On-label devices** | **Off-label SE** | **Off-label BE** |  | |
| **Outcomes** | **n** | **N** | **Event rate (95% CI)** | **Event rate (95% CI)** | **Event rate (95% CI)** | **χ^2^** | ***P**** |
| all cause mortality in hospital | 9 | 775 | 4.0% (1.2–12.1%) | 7.1% (4.7–10.7%) | 3.9% (2.1–7.1%) | 2.98 | 0.225 |
| SVI | 14 | 1014 | 3.1% (1.5–6.5%) | 12.0% (8.4–16.9%) | 5.4% (3.3–8.8%) | 13.89 | 0.001¡£ |
| Moderate or severe AR in hospital | 14 | 1076 | 1.8% (0.8–3.6%) | 7.2% (4.7–10.9%) | 9.2% (6.2–13.6%) | 15.75 | 0.000¡¢ |
| Device success | 10 | 786 | 94.9% (89.8–97.5%) | 86.3% (74.2–93.2%) | 89.4% (67.8–97.1%) | 3.97 | 0.138 |
|  |  |  | **SA-3 excluding European cohorts** | | |  |  |
| all cause mortality in 1 year | 10 | 701 | 8.6% (4.2–16.7%) | 5.7% (2.9–11.0%) | 8.4% (5.6–12.6%) | 1.09 | 0.580 |
| all cause mortality in 30 days | 12 | 928 | 2.9% (1.5–5.6%) | - | 6.8% (3.7–12.2%) | 3.39 | 0.066 |
| all cause mortality in hospital | 16 | 1358 | 2.1% (1.2–3.6%) | 5.9% (3.8–8.9%) | 3.9% (2.1–7.1%) | 8.29 | 0.016¡ |
| SVI | 19 | 1393 | 2.3% (1.0–5.0%) | 18.2% (13.4–24.1%) | 5.5% (2.9–10.2%) | 31.23 | 0.000¡£ |
| Moderate or severe AR in hospital | 22 | 1728 | 2.6% (1.6–4.1%) | 5.7% (3.6–9.0%) | 9.3% (6.3–13.6%) | 17.42 | 0.000¡¢ |
| Device success | 15 | 1176 | 94.5% (90.9–96.7%) | 81.2% (74.6–86.5%) | 89.4% (67.8–97.1%) | 16.39 | 0.000¡ |
|  |  |  | **SA-3 excluding North American cohorts** | | |  |  |
| all cause mortality in 1 year | 11 | 598 | 5.2% (1.2–19.1%) | 6.5% (3.6–11.8%) | 9.9% (6.4–14.9%) | 1.72 | 0.424 |
| all cause mortality in 30 days | 15 | 861 | 3.5% (1.8–6.9%) | 4.4% (2.1–8.9%) | 6.1% (3.2–11.3%) | 1.40 | 0.497 |
| all cause mortality in hospital | 18 | 1321 | 2.7% (1.6–4.8%) | 4.5% (2.7–7.3%) | 3.9% (2.1–7.1%) | 1.74 | 0.418 |
| SVI | 23 | 1668 | 1.8% (1.1–3.1%) | 17.9% (13.5–23.4%) | 5.4% (3.3–8.8%) | 63.19 | 0.000¡¢£ |
| Moderate or severe AR in hospital | 24 | 1567 | 2.5% (1.6–4.1%) | 5.7% (3.4–9.4%) | 9.2% (6.2–13.6%) | 16.37 | 0.000¡¢ |
| Device success | 18 | 1167 | 94.4% (91.3–96.4%) | 79.2% (73.4–84.1%) | 89.4% (67.8–97.1%) | 26.48 | 0.000¡ |
| n:Number of Studies; N:Overall Sample Size; SA: sensitivity analyses; ¡ On-label devices vs Off-label SE; ¢ On-label devices vs Off-label BE;  £ Off-label SE vs Off-label BE; * Test for subgroup differences; SA-3, leave-region-out analyses excluding European, or North American cohorts | | | | | | | |
| Supplemental Table 6 (continued) | | | | | | | |
|  |  |  | **On-label devices** | **Off-label SE** | **Off-label BE** |  |  |
| **Outcomes** | **n** | **N** | **Event rate (95% CI)** | **Event rate (95% CI)** | **Event rate (95% CI)** | **χ^2^** | ***P**** |
|  |  |  | **SA-4** | | |  |  |
| all cause mortality in 1 year | 11 | 761 | 6.7% (2.9–14.7%) | 5.7% (2.9–11.0%) | 9.9% (6.4–14.9%) | 2.14 | 0.344 |
| all cause mortality in 30 days | 14 | 1122 | 3.2% (1.7–6.1%) | 6.1% (2.7–13.4%) | 6.1% (3.2–11.3%) | 2.35 | 0.309 |
| all cause mortality in hospital | 17 | 1419 | 2.5% (1.3–4.6%) | 5.9% (3.8–8.9%) | 3.9% (2.1–7.1%) | 5.14 | 0.077 |
| SVI | 21 | 1624 | 1.8% (1.1–3.1%) | 18.8% (13.9–25.0%) | 5.4% (3.3–8.8%) | 62.74 | 0.000¡¢£ |
| Moderate or severe AR in hospital | 25 | 1857 | 2.3% (1.4–3.7%) | 5.7% (3.6–9.0%) | 9.2% (6.2–13.6%) | 20.03 | 0.000¡¢ |
| Device success | 17 | 1272 | 94.4% (91.3–96.4%) | 80.6% (73.6–86.2%) | 89.4% (67.8–97.1%) | 19.8 | 0.000¡ |

n:Number of Studies; N:Overall Sample Size; SA: sensitivity analyses; ¡ On-label devices vs Off-label SE; ¢ On-label devices vs Off-label BE; £ Off-label SE vs Off-label BE; * Test for subgroup difference; SA-4, excluding small studies (< 30 patients) reporting a single valve platform


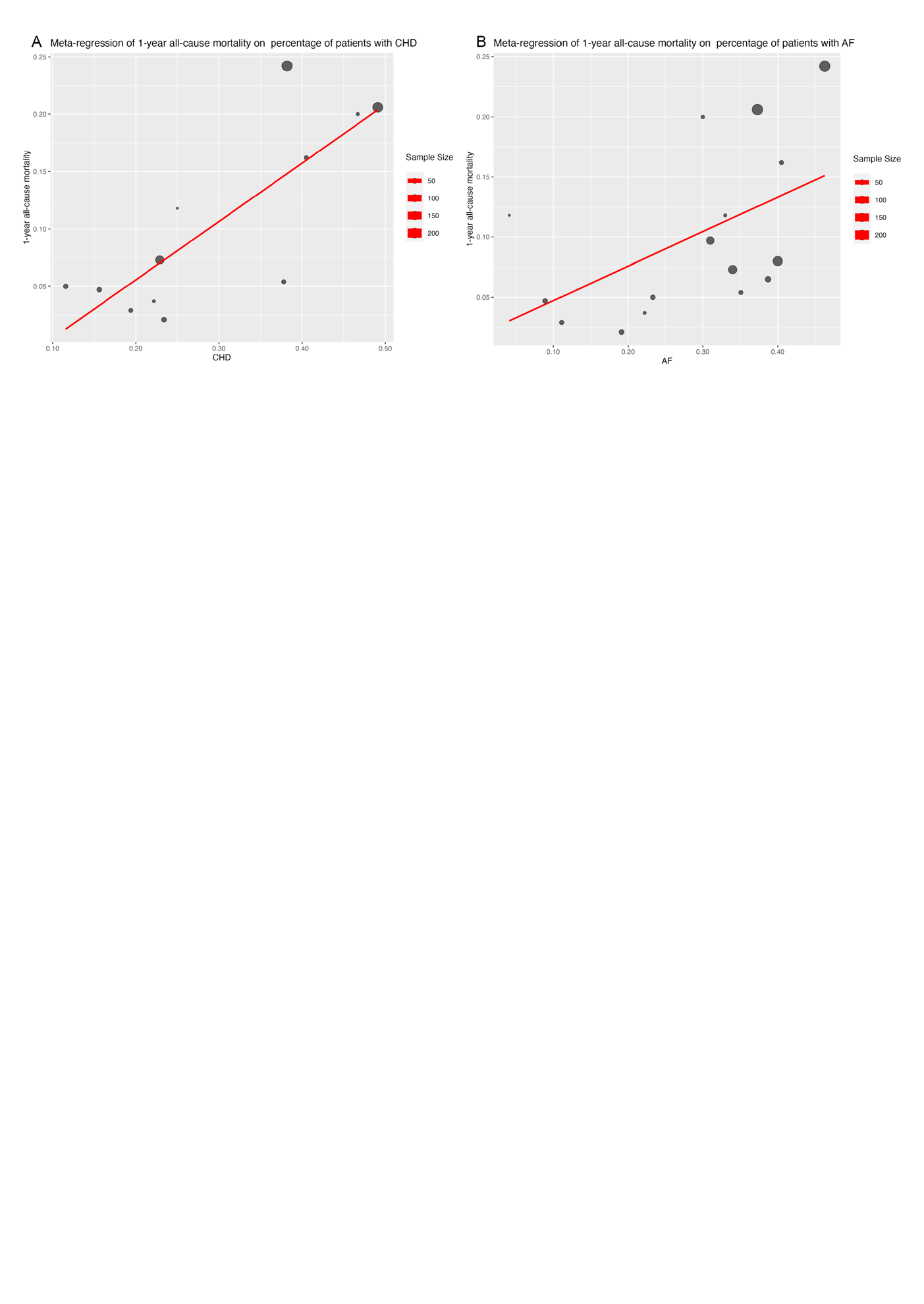
Supplementary Figure 1 Meta-regression of 1-year all-cause mortality.


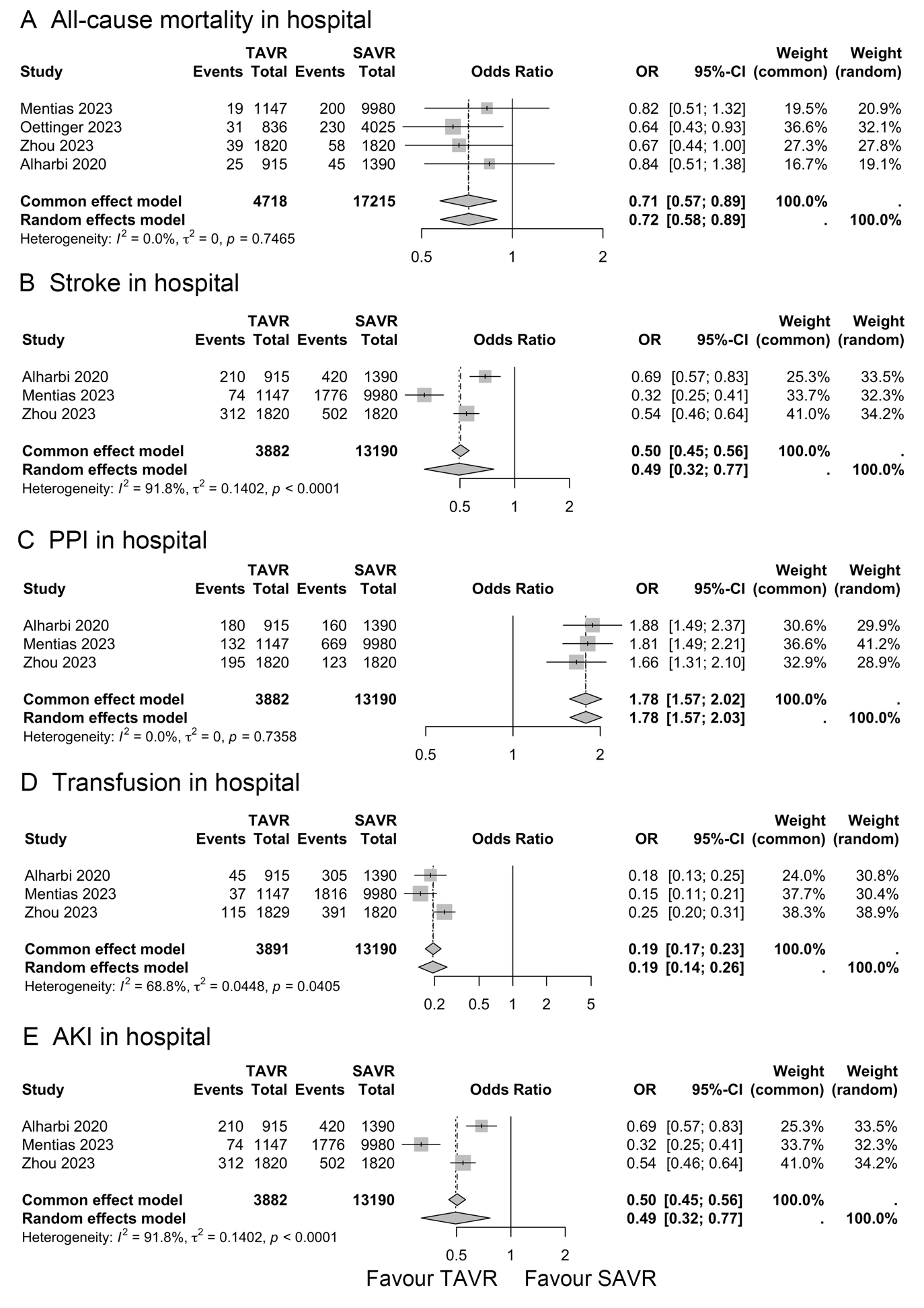


Supplementary Figure 2 Forest Plot of In-Hospital Outcomes: TAVR vs. SAV

Supplementary Table 7 Summary of the studies between SAVR and TAVR

| **First Author & Year** | **Study Design** | **Country** | **Group** | **Sample Size** | **Age (yrs)** | **Female (%)** | **HTN (%)** | **DM (%)** | **CAD (%)** | **AF (%)** | **COPD (%)** | **Peripheral Arterial Disease (%)** |
| --- | --- | --- | --- | --- | --- | --- | --- | --- | --- | --- | --- | --- |
| **Mentias, 2023** | Propensity matched retrospective study | USA | TAVR | 1147 | 76.9 ± 7.1 | 40 | 85.0 | 27.0 | 60.0 | 28.0 | - | 26.0 |
| **Mentias, 2023** | Propensity matched retrospective study | USA | SAVR | 9980 | 72.9 ± 5.1 | 40 | 85.0 | 27.0 | 60.0 | 28.0 | - | 26.0 |
| **Oettinger, 2023** | Retrospective analysis | Germany | TAVR | 836 | 76.8 ± 8.8 | 34.5 | 40.0 | 18.7 | 46.2 | 47.1 | 18.8 | 8.3 |
| **Oettinger, 2023** | Retrospective analysis | Germany | SAVR | 4025 | 62.8 ± 13.6 | 25.4 | 58.0 | 12.2 | 14.6 | 44.5 | 7.2 | 2.8 |
| **Zhou，2023** | Propensity matched retrospective study | USA | TAVR | 1820 | 70.5 ± 12.5 | 36 | - | 15.8 | - | - | 26.7 | 25.5 |
| **Zhou，2023** | Propensity matched retrospective study | USA | SAVR | 1820 | 70.0 ± 10.4 | 35.7 | - | 15.8 | - | - | 26.9 | 24.1 |
| **Alharbi，2020** | Propensity matched retrospective study | USA | TAVR | 915 | 78 (IQR: 68–85) | 29.0 | 44.8 | 27.4 | 62.8 | - | 25.1 | 21.9 |
| **Alharbi，2020** | Propensity matched retrospective study | USA | SAVR | 1390 | 74 (IQR: 67–80) | 28.8 | 40.3 | 23.0 | 59.7 | - | 23.0 | 25.2 |


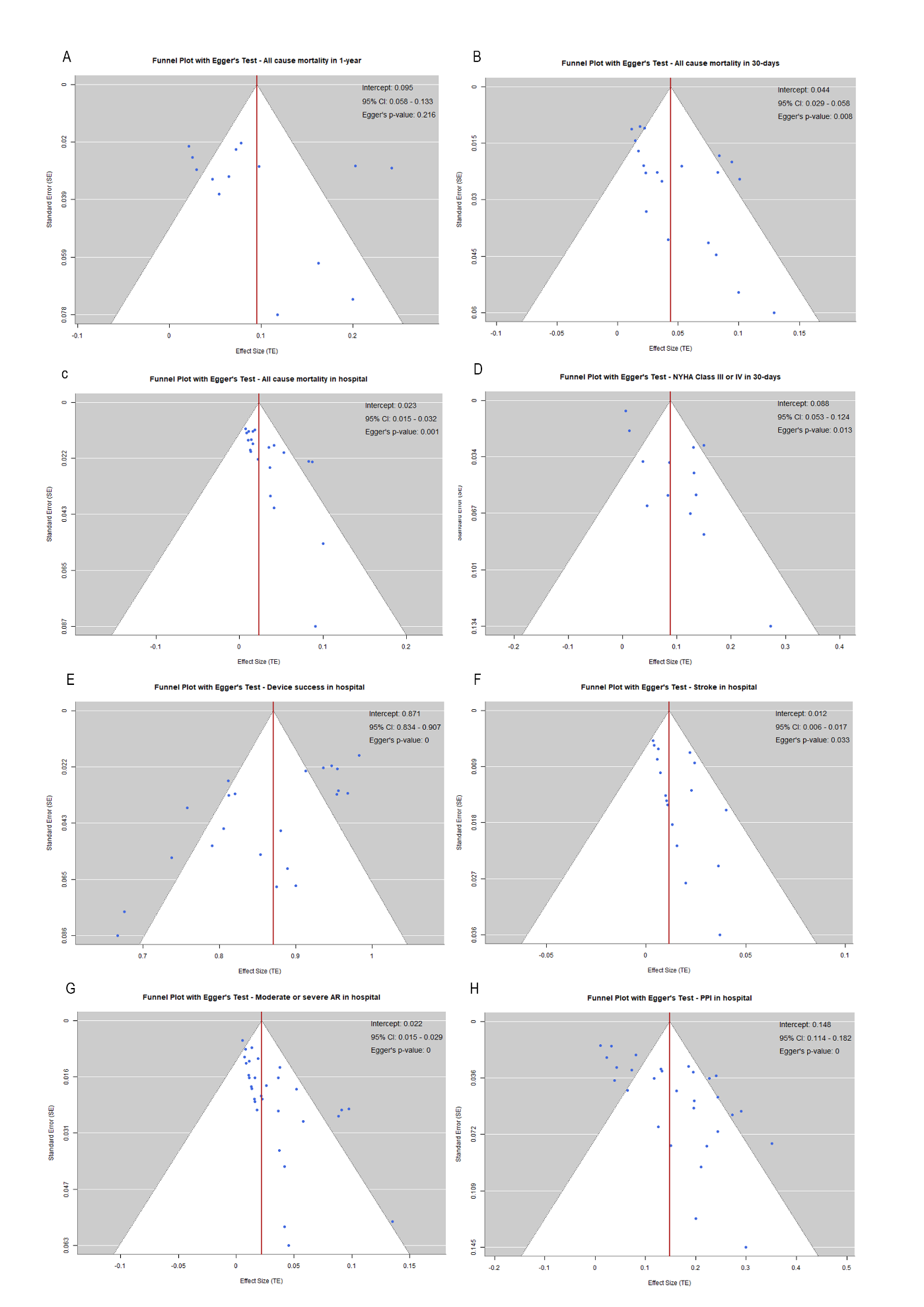
 Supplementary Figure 3 Publication Bias Assessment

Supplementary Table 8 MINORS Criteria.

| Author et al. | 1 | 2 | 3 | 4 | 5 | 6 | 7 | 8 | Total |
| --- | --- | --- | --- | --- | --- | --- | --- | --- | --- |
| Yang et al 2024 | 2 | 2 | 2 | 2 | 1 | 1 | 2 | 1 | 13 |
| Yu et al 2024 | 2 | 2 | 0 | 2 | 2 | 2 | 2 | 0 | 12 |
| Vahl et al 2024 | 2 | 2 | 2 | 2 | 2 | 2 | 2 | 2 | 16 |
| Tsai et al 2024 | 2 | 2 | 0 | 2 | 2 | 1 | 1 | 0 | 10 |
| Poletti et al 2024, BE-PANTHEON | 2 | 2 | 0 | 2 | 2 | 2 | 2 | 0 | 12 |
| Poletti et al 2024, PURPOSE | 2 | 2 | 0 | 2 | 2 | 2 | 2 | 0 | 12 |
| Mao et al 2024 | 2 | 2 | 2 | 2 | 2 | 2 | 2 | 0 | 14 |
| Le Ruz et al 2024 | 2 | 2 | 2 | 2 | 2 | 2 | 2 | 0 | 14 |
| Kong et al 2024 | 2 | 2 | 2 | 2 | 2 | 2 | 2 | 0 | 14 |
| Hinkov et al 2024 | 2 | 2 | 0 | 2 | 2 | 2 | 2 | 0 | 12 |
| Zheng et al 2023 | 2 | 2 | 2 | 2 | 2 | 2 | 2 | 0 | 14 |
| Sánchez-Luna et al 2023 | 2 | 2 | 2 | 2 | 2 | 2 | 2 | 0 | 14 |
| Poletti et al 2023 PANTHEON | 2 | 2 | 0 | 2 | 2 | 2 | 2 | 0 | 12 |
| Jin et al 2024 | 2 | 2 | 0 | 2 | 2 | 2 | 2 | 0 | 12 |
| Garcia et al. 2024 | 2 | 2 | 2 | 2 | 2 | 2 | 2 | 0 | 14 |
| Delhomme et al 2023 | 2 | 2 | 1 | 2 | 2 | 2 | 2 | 1 | 14 |
| Chen et al. 2023 | 2 | 2 | 2 | 2 | 2 | 2 | 2 | 0 | 14 |
| Adam et al. 2023 | 2 | 2 | 2 | 2 | 2 | 2 | 2 | 2 | 16 |
| Wang et al. 2022 | 2 | 2 | 2 | 2 | 2 | 2 | 2 | 0 | 14 |
| Liu et al 2022 | 2 | 2 | 2 | 2 | 2 | 2 | 2 | 2 | 16 |
| Kong et al 2022 | 2 | 2 | 1 | 2 | 2 | 2 | 0 | 1 | 12 |
| Chen et al. 2022 | 2 | 2 | 0 | 2 | 2 | 1 | 0 | 1 | 10 |
| Purita et al 2020 | 2 | 2 | 2 | 2 | 2 | 2 | 2 | 2 | 16 |
| Liu et al 2020 | 2 | 2 | 0 | 2 | 2 | 2 | 2 | 1 | 13 |
| Anwaruddin et al 2019 | 2 | 2 | 0 | 2 | 2 | 2 | 2 | 2 | 14 |
| Tung et al 2018 | 2 | 2 | 2 | 2 | 2 | 2 | 2 | 2 | 16 |
| Seiffert et al 2014 | 2 | 2 | 0 | 2 | 2 | 2 | 2 | 1 | 13 |
| Silaschi et al 2018 | 2 | 2 | 2 | 2 | 2 | 2 | 2 | 2 | 16 |
| De Backer et al 2018 | 2 | 2 | 2 | 2 | 2 | 2 | 2 | 2 | 16 |
| Sawaya et al 2017 | 2 | 2 | 2 | 2 | 2 | 2 | 2 | 2 | 16 |
| Toggweiler et al 2018 | 2 | 2 | 0 | 2 | 2 | 2 | 2 | 1 | 13 |
| Yoon et al 2017 | 2 | 2 | 2 | 2 | 2 | 2 | 2 | 2 | 16 |
